# Supplementary material for: Sustained TNF-α stimulation leads to transcriptional memory that greatly enhances signal sensitivity and robustness
Source: eLife. 2020 Nov 6;9:e61965. doi: 10.7554/eLife.61965 (PMC7704108; doi:10.7554/eLife.61965)
Supplement: Supplementary file 1. [file elife-61965-supp1.docx]

Supplementary File 1. Oligonucleotides

| gRNA sequences for genome engineering | |
| --- | --- |
| MER11B-right | TTGTTCGACTCCCTGACGAC |
|  | GCCCGCCACTCCAAGTTCTG |
| MER11B-left | CTGGGAATTGTGCCATGCTA |
|  | CGAACAAGCCTTCATTGCCA |
| ERV | GCGTGCGGGGATTCCAGTCT |
|  | GCCCGCCACTCCAAGTTCTG |
|  |  |
| Primers for verification of ERV, MER11B-left, MER11B-right KO cell lines | |
| ERV WT-F | CTAAAAATTTGCGAGGTGGG |
| ERV WT-R | TGGACAGATGGCCATACTGC |
| ERV KO-F | GGGTGAACTTATTGTCTGAC |
| ERV KO-R | AGTGGACAGGCAAAGTTTGG |
| MER11B-left WT-F | TGGTGTCTGGTAAGCACTCG |
| MER11B-left WT-R | GCTGCTGACATGTGATGTCT |
| MER11B-left KO-F | ACACTGTGCAAAGACGCTAG |
| MER11B-left KO-R | GGAAATGGTCAAAGGACACC |
| MER11B-right WT-F | GAGGTGACATACATCCTCAG |
| MER11B-rght WT-R | AACCTTTGGAACTGGCACTC |
| MER11B-right KO-F | CCCCAAACAGTGATGGGAAC |
| MER11B-right KO-R | CAGCCTCTGATAACCATCTC |
|  |  |
| RT-qPCR primers | |
| *CALCB*-F | CGGCCACACTCAGTAAAGAG |
| *CALCB*-R | TCACACAGGTGGCAGTGTTG |
| *EGFP*-F | AAGCTGACCCTGAAGTTCATCTGC |
| *EGFP*-R | CTTGTAGTTGCCGTCGTCCTTGAA |
| *GAPDH*-F | CTGGGCTACACTGAGCACC |
| *GAPDH*-R | AAGTGGTCGTTGAGGGCAATG |
| *PTGES*-F | TCCTAACCCTTTTGTCGCCTG |
| *PTGES*-R | CGCTTCCCAGAGGATCTGC |
|  |  |
| Primers for locus-specific amplification from bisulfite-treated DNA | |
| MER11B-right-F1 | GTAAATGGAGGTAGGGTAAGATTAT |
| MER11B-right-R1 | TCTCCAAAAACCAAATCTATATCAC |
| MER11B-right-F2 | AGGATTAGGGTGAAATTAAAATTGT |
| MER11B-right-R2 | ATATTTAAACAAATAACCATACTAC |
| MER11B-right-F3 | AGGATTAGGGTGAAATTAAAATTGT |
| MER11B-right-R3 | TAAATCTCTAAAATAACCACTCTAA |
| MER11B-left-F1 | TATGAATTGTTTATAAGTATGTGTG |
| MER11B-left-R1 | CACCCAATACATACCTATAAAACAA |
| MER11B-left-F2 | TTGGGTATTTTGAAAAAAGAATAGG |

| MER11B-left-R2 | TCTAAAAATTATACCATACTATAAC |
| --- | --- |
| MER11B-left-F3 | TTTTAGGTGTAAGTTTTTAAAATGG |
| MER11B-left-R3 | AACAAAATAAAATCACAAAACCAAA |
| CMV-F1 | GTTTAGTATATGATTTTATGGG |
| CMV-R1 | CCAAAATAAACACCACCCC |
| CMV-F2 | ATTTTTTTATTTGGTAGTATATTTA |
| CMV-R2 | CCCTTACTCACCATAATAAC |
|  |  |
| Methylated Adaptor sequence (All the cytosines are methylated) | |
| sequence1 | 5’-ACACTCTTTCCCTACACGACGCTCTTCCGATC-s-T-3’ |
| sequence2 | 5’-  /5Phos/GATCGGAAGAGCACACGTCTGAACTCCAGTC-3’ |
