# Supplementary material for: Sustained TNF-α stimulation leads to transcriptional memory that greatly enhances signal sensitivity and robustness
Source: eLife. 2020 Nov 6;9:e61965. doi: 10.7554/eLife.61965 (PMC7704108; doi:10.7554/eLife.61965)
Supplement: Supplementary file 2. [file elife-61965-supp2.docx]

Supplementary File 2. Genomic Sequences for Locus-Specific Bisulfite PCR Sequencing Analysis

| Genomic Sequences for Locus-Specific Bisulfite PCR Sequencing Analysis | |
| --- | --- |
| CMV promoter | TTGGCAGTACATCTACGTATTAGTCATCGCTATTACCATG GTGATGCGGTTTTGGCAGTACATCAATGGGCGTGGATAG CGGTTTGACTCACGGGGATTTCCAAGTCTCCACCCCATTG ACGTCAATGGGAGTTTGTTTTGGCACCAAAATCAACGGG ACTTTCCAAAATGTCGTAACAACTCCGCCCCATTGACGCA AATGGGCGGTAGGCGTGTACGGTGGGAGGTCTATATAAG  CAGAGCTGGTTTAGTGAACCGTCAGATC |
| MER11B-left | CGCTCTGGGAATGTCTGTCTTTTACGGTTGAAGATAAGGG ATGAAATAAGCTTTGGTCTCCCGTAGCATTCCCAGGCCTA TTAGGACGAGGAAATTCCCACCTAGTAAATTTTTAGTCAG ACCGGTTGTCTGCTTTCAAATCCTGTCTCCTGATAAGTTGT TATCAATGACAATGCGTGCCCAAAACTTCATTAGCAATTT TAATTTCG |
| MER11B-right | CGCATTGTCATTGATAACATCTTATCAGGAGACAGGGTTT GAAAGCAGACAATCGGTCTAACTAAAAATTTGCGAGGTG GGAATTTCCTTGTCCTAATAGGCCTGGGAACGCTACGGGA GACCGAGGCTTATTTCATCCCTTATCTTCAACCATAAAAG ACAGACG |
